# Targeting SERCA2 in organotypic epidermis reveals MEK inhibition as a therapeutic strategy for Darier disease

**DOI:** 10.1172/jci.insight.170739

**Published:** 2023-09-22

**Authors:** Shivam A. Zaver, Mrinal K. Sarkar, Shaun Egolf, Jonathan Zou, Afua Tiwaa, Brian C. Capell, Johann E. Gudjonsson, Cory L. Simpson

**Affiliations:** 1 Division of Dermatology, Department of Medicine, and; 2 Medical Scientist Training Program, University of Washington, Seattle, Washington, USA.; 3 Department of Dermatology, University of Michigan, Ann Arbor, Michigan, USA.; 4 Department of Dermatology, University of Pennsylvania Perelman School of Medicine, Philadelphia, Pennsylvania, USA.; 5 Institute for Stem Cell and Regenerative Medicine, University of Washington, Seattle, Washington, USA.

**Keywords:** Dermatology, Cell migration/adhesion, Genetic diseases, Skin

## Abstract

Mutation of the *ATP2A2* gene encoding sarco-endoplasmic reticulum calcium ATPase 2 (SERCA2) was linked to Darier disease more than 2 decades ago; however, there remain no targeted therapies for this disorder causing recurrent skin blistering and infections. Since *Atp2a2*-knockout mice do not phenocopy its pathology, we established a human tissue model of Darier disease to elucidate its pathogenesis and identify potential therapies. Leveraging CRISPR/Cas9, we generated human keratinocytes lacking SERCA2, which replicated features of Darier disease, including weakened intercellular adhesion and defective differentiation in organotypic epidermis. To identify pathogenic drivers downstream of SERCA2 depletion, we performed RNA sequencing and proteomics analysis. SERCA2-deficient keratinocytes lacked desmosomal and cytoskeletal proteins required for epidermal integrity and exhibited excess MAPK signaling, which modulates keratinocyte adhesion and differentiation. Immunostaining patient biopsies substantiated these findings, with lesions showing keratin deficiency, cadherin mislocalization, and ERK hyperphosphorylation. Dampening ERK activity with MEK inhibitors rescued adhesive protein expression and restored keratinocyte sheet integrity despite SERCA2 depletion or chemical inhibition. In sum, coupling multiomic analysis with human organotypic epidermis as a preclinical model, we found that SERCA2 haploinsufficiency disrupts critical adhesive components in keratinocytes via ERK signaling and identified MEK inhibition as a treatment strategy for Darier disease.

## Graphical abstract

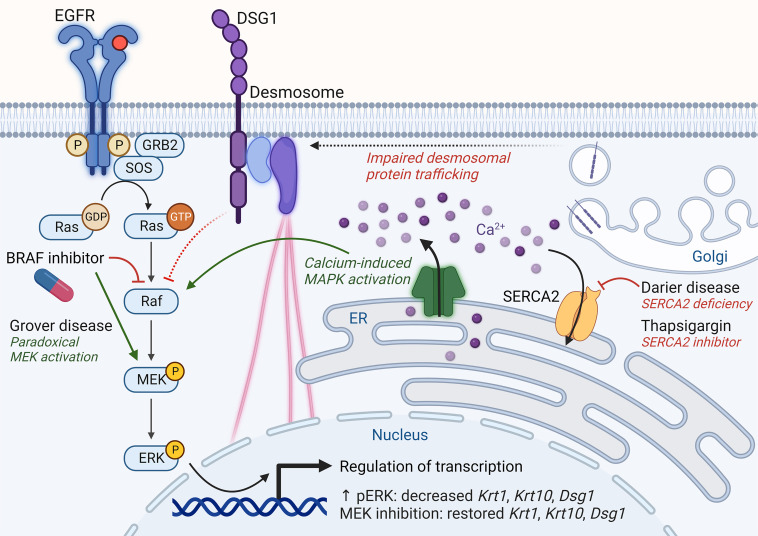

## Introduction

Though advances in gene sequencing have facilitated the diagnosis of rare inherited disorders and enhanced our understanding of their pathogenic mechanisms (1–3), most genetic skin diseases lack proven, rational molecular therapies (4, 5). Mutation of the *ATP2A2* gene, which encodes sarco-endoplasmic reticulum calcium ATPase 2 (SERCA2), causes Darier disease (DD) (6, 7), a dermatologic disorder characterized by aberrant epidermal differentiation and impaired keratinocyte adhesion via desmosomes (8, 9), which manifests as recurrent skin blisters, erosions, and infections (10–13). Despite knowing the genetic etiology of this disease for more than 20 years, there are no FDA-approved therapies for DD, and no drugs that directly target the pathogenic effects of SERCA2 deficiency have advanced to prospective clinical trials (14).

While systemic retinoids have been used off-label for treatment of DD (15, 16), their long-term toxicity and highly teratogenic nature limit utilization in patients with this lifelong disorder (17, 18). Similarly, topical corticosteroids and antimicrobials can treat secondary inflammation and infection in localized lesions (14) but are not specifically aimed at the underlying disease pathophysiology and exhibit limited efficacy, especially in widespread disease that can lead to serious illness (12, 13, 19). Unfortunately, targeted ablation of the *Atp2a2* gene in mice did not replicate DD pathology but instead led to increased age-related keratinocyte carcinomas (20, 21), a risk not borne out in patients.

We leveraged cultured human keratinocytes and organotypic epidermis (22) to establish a preclinical model of DD to better elucidate its pathogenesis and identify potential therapeutic avenues to help direct future clinical trials. Using CRISPR/Cas9 gene editing (23, 24), we generated human keratinocytes having the first exon of *ATP2A2* disrupted and performed multiomics analysis, which revealed inhibition of the MAPK pathway as a treatment strategy for DD.

## Results

### Loss of SERCA2 in human keratinocytes impairs cytosolic calcium handling and reduces intercellular adhesive strength.

DD, an autosomal dominant disorder, has been linked to various *ATP2A2* gene mutations (6, 11, 25), most of which are predicted to cause haploinsufficiency of SERCA2, a calcium channel localized to the endoplasmic reticulum (ER) (26). SERCA2 function is essential for calcium-dependent assembly of intercellular junctions, particularly desmosomes, which maintain epidermal integrity (8, 9). Also, SERCA2 controls levels of cytosolic calcium, a master regulator of the terminal phase of keratinocyte differentiation (27, 28), termed cornification, in which cells in the upper epidermis degrade their nuclei and organelles to form the flattened, outermost layers of the cutaneous barrier (29, 30). In patients with DD, the loss of intercellular adhesion and dysfunctional keratinocyte differentiation manifest as blistering, thick scaling, and fissuring of the skin (10, 26).

**Figure 1:**
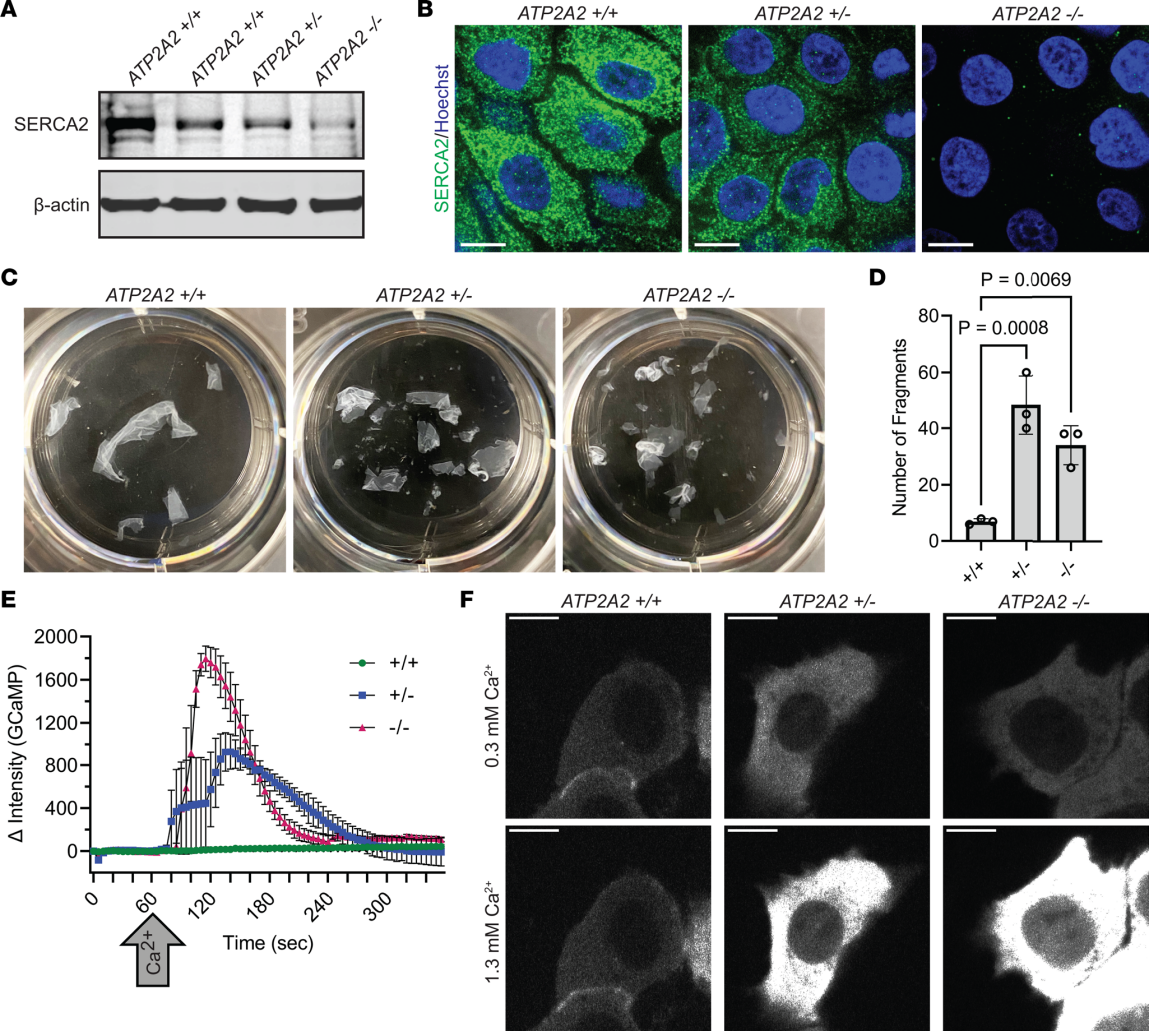
Loss of SERCA2 in human keratinocytes impairs cytosolic calcium handling and reduces intercellular adhesive strength. (**A**) Immunoblot of SERCA2 in lysates from *ATP2A2* wild-type (WT, +/+), heterozygous (HET, +/-), and homozygous knockout (KO, -/-) cells. hTERT-immortalized human epidermal keratinocytes (THEKs) were differentiated in E-medium for 72 hours before lysate harvesting; data represent 3 independent experiments; and β-actin is a loading control. (**B**) Immunofluorescence of SERCA2 (green) in WT, HET, and KO THEKs; images are representative of 14 independent high-powered fields (hpf) per genotype; Hoechst (blue) stains nuclei; scale bar = 10 μm. (**C**) Mechanical dissociation of monolayers from control (+/+), HET (+/-), and KO (-/-) cells grown in 1.3 mM CaCl_2_ for 72 hours prior to using dispase to release intact monolayers; representative images of fragmented monolayers transferred into 6-well cell culture plates after mechanical stress are shown. (**D**) Graphs display mean ± SD of the number of epithelial fragments with data plotted for *N* = 3 biological replicates; *P* values from 1-way ANOVA with Dunnett’s adjustment for multiple comparisons. (**E**) Change (Δ) in intensity of GCaMP in control (+/+), HET (+/-), and KO (-/-) THEKs from baseline at *t* = 0 seconds with addition of 1 mM CaCl_2_ at *t* = 60 seconds (arrow); data plotted as mean ± SEM from *N* = 3 independent experiments per genotype. (**F**) Representative fluorescence images of GCaMP in WT, HET, and KO cells in low CaCl_2_ (left) or high CaCl_2_ (right); scale bar = 10 μm.

Since *Atp2a2*-KO mice did not phenocopy DD (20), preclinical studies of its pathogenesis have been limited and no therapies have advanced to clinical trials to achieve FDA approval (14). Given the cutaneous pathology of DD is limited to the epidermis, we used CRISPR/Cas9 to ablate the *ATP2A2* gene in keratinocytes (24), which can be grown into a fully stratified epidermis within an organotypic model (22). We targeted the first exon of *ATP2A2* (or a pseudogene as a control) in hTERT-immortalized human epidermal keratinocytes (THEKs) from the parental line N/TERT-2G (31); this allowed isolation of stable homozygous knockout (KO) and heterozygous (HET) cell lines harboring frameshift mutations that depleted SERCA2 as verified by immunoblotting cell lysates and immunostaining fixed cells (Figure 1, A and B). SERCA2-deficient cells displayed similar proliferation rates to control cells (Supplemental Figure 1; supplemental material available online with this article; https://doi.org/10.1172/jci.insight.170739DS1).

Given the essential role of SERCA2 in assembling cell-cell junctions (8, 9), we assessed the mechanical integrity of control and SERCA2-deficient THEKs using an established dispase-based assay to quantify intercellular adhesive strength of epithelial cell monolayers (32, 33). We found that both HET and KO THEKs exhibited reduced integrity, reflected by increased fragmentation of epithelial sheets upon mechanical stress (Figure 1, C and D). We also used GCaMP, a ratiometric, green fluorescent protein–based (GFP-based), genetically encoded calcium indicator (34, 35), to verify that depletion of the ER-localized calcium ATPase altered cytosolic calcium handling. THEKs were transduced with GCaMP, then imaged by live microscopy during exposure to increased extracellular calcium. While control cells demonstrated minimal change in GCaMP intensity after treatment with calcium, KO cells experienced large increases in cytosolic calcium; HET cells exhibited an intermediate phenotype, as would be expected with haploinsufficiency of SERCA2-driven transport of calcium into the ER (Figure 1, E and F).

### Keratinocytes lacking SERCA2 exhibit transcriptomic alterations in mediators of epidermal adhesion and differentiation.

**Figure 2:**
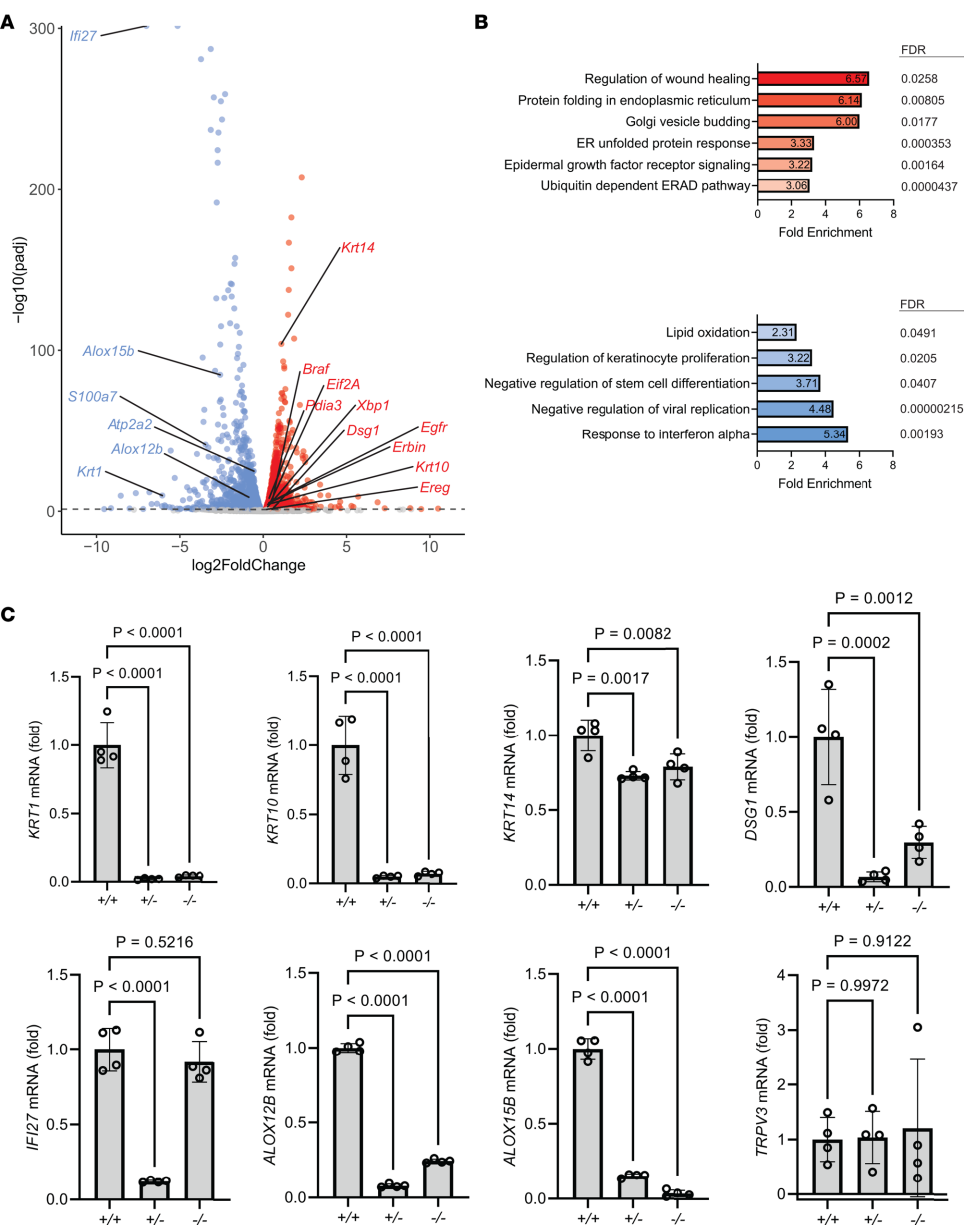
Keratinocytes lacking SERCA2 exhibit transcriptomic alterations in mediators of epidermal adhesion and differentiation. (**A**) Results from bulk RNA sequencing of SERCA2 HET versus control cells. Volcano plot depicts log_2_ fold-changes of genes significantly downregulated (blue) and upregulated (red) with cutoff (dashed line) 0.05 for adjusted *P* value. (**B**) Gene ontology (GO) analysis of transcripts significantly altered (adjusted *P* ≤ 0.05) in HET cells revealed upregulation (red) in genes controlling ER stress and growth factor signaling and downregulation (blue) of genes controlling epidermal differentiation and antiviral response. (**C**) Control (+/+), HET (+/-), or KO (-/-) THEKs were differentiated in E-medium for 24 hours, and mRNA transcripts were quantified by reverse transcription quantitative PCR (RT-qPCR). Graphs display mean ± SD with plotted values from *N* = 4 biological replicates; *P* values from 1-way ANOVA with Dunnett’s adjustment for multiple comparisons. DSG1, desmoglein 1; KRT, keratin.

To identify potential pathogenic drivers of DD in an unbiased manner, we assessed downstream consequences of SERCA2 depletion on the transcriptome in HET and KO keratinocytes. Bulk RNA sequencing (RNA-Seq) revealed that HET cells exhibited significant changes in mRNA transcript profiles (Figure 2A), with Gene Ontology (GO) analysis identifying alterations in gene subsets linked to intercellular adhesion, calcium regulation, and epidermal differentiation (Figure 2B), consistent with known pathologic features of DD. However, we also identified a gene signature indicating elevation in EGFR signaling and the downstream MAPK pathway, which are known to regulate epidermal differentiation and adhesion (36–40). We verified these SERCA2 deficiency–related changes in gene expression with quantitative reverse transcription PCR (RT-qPCR) (Figure 2C).

Relative to controls, HET keratinocytes showed marked reduction in mRNA encoding KRT1 and KRT10 intermediate filament cytoskeletal proteins and the desmosomal cadherin DSG1, all of which are known to exhibit differentiation-dependent expression in the suprabasal epidermal layers (41). In contrast, the effect of SERCA2 deficiency on mRNA encoding the primary keratin of the basal epidermal layer, KRT14, was much smaller. While GO analysis identified changes in some differentiation-associated calcium-binding proteins, the mRNA encoding TRPV3, a plasma membrane calcium channel recently linked to calcium influx during the final stage of keratinocyte differentiation (30), was unchanged in HET or KO cells. Interestingly, some genes exhibited disparate effects in KO versus HET lines, such as the interferon response gene *IFI27*, which was only depleted in HET cells.

The expression of lipoxygenase genes that drive membrane lipid modifications essential for terminal keratinocyte differentiation and epidermal barrier formation (42, 43) was also significantly reduced in SERCA2-deficient cells. Loss of ALOX12B, in particular, has been associated with autosomal recessive congenital ichthyosis (ARCI) (44), a group of inherited dermatologic diseases characterized by skin scaling and epidermal barrier defects due to defective keratinocyte maturation (43, 45), which shares clinical features with DD, including response to retinoid therapy (17).

### Proteomics analysis of SERCA2-deficient keratinocytes reveals alterations in key regulators of tissue integrity and skin barrier formation.

**Figure 3:**
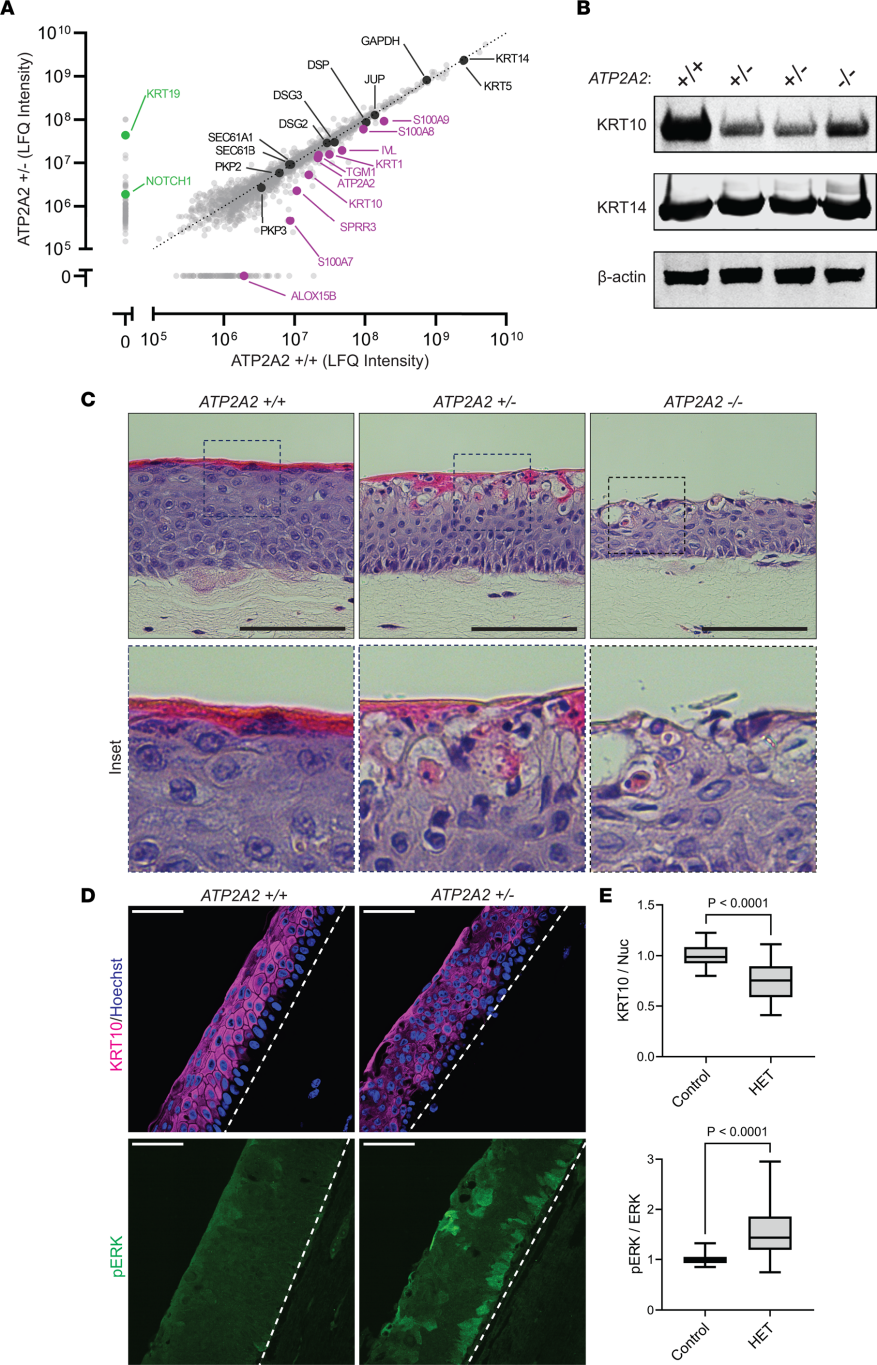
Proteomics analysis of SERCA2-deficient keratinocytes reveals alterations in key regulators of tissue integrity and skin barrier formation. (**A**) Label-free quantitative (LFQ) mass spectrometry–based comparison of the proteomes of control versus HET THEKs differentiated in E-medium for 72 hours. Proteins highlighted in purple were absent or detected in lower abundance in HET cells; proteins highlighted in green were detected in higher abundance in control THEKs; proteins highlighted in black were detected in similar abundance in control and HET cells; raw values and statistical analysis summarized in Supplemental Table 1. (**B**) Immunoblotting of KRT10 and KRT14 (on a separate blot run in parallel) in lysates from control, HET, and KO THEKs differentiated in E-medium for 72 hours; data representative of 5 independent experiments; β-actin is a loading control. (**C**) H&E-stained tissue cross sections from organotypic cultures grown from control, HET, or KO THEKs. HET and KO cultures display aberrant differentiation with the upper granular layers exhibiting vacuolization and loss of cohesion along with dyskeratotic cells having deeply pink cytoplasm and condensed nuclei indicative of aberrant cornification (insets); scale bar = 100 μm; insets, original magnification = 40×. (**D**) Immunostaining of KRT10 and p-ERK in tissue cross sections from organotypic cultures of control or HET THEKs; scale bar = 50 μm; dashed line marks bottom of epidermis. (**E**) Quantification of tissue immunostaining of KRT10 (relative to Hoechst) and p-ERK (relative to total ERK); data shown as a box plot of the 25th–75th percentile with a line at the median from *N* ≥ 26 nonoverlapping hpf per condition from 4 experiments using 2 independent HET lines; control mean normalized to 1; *P* values from 2-tailed unpaired Student’s *t* test. DSP, desmoplakin; JUP, plakoglobin; IVL, involucrin; TGM1, transglutaminase 1; PKP, plakophilin; p-, phosphorylated.

To determine if transcriptional changes in SERCA2-deficient keratinocytes translated to effects on protein expression, we performed label-free mass spectrometry–based (MS-based) identification of the proteome in 2 independent HET cell lines (Figure 3A and Supplemental Table 1). This technique verified deficiency of SERCA2 itself, while the levels of other ER-localized proteins (SEC61B, SEC61A1) and an often-used housekeeping gene (GAPDH) were not significantly changed in HET cells compared to controls.

Proteomics analysis again revealed substantial reduction in proteins that drive membrane lipid modifications (ALOX15B) and several members of the S100 family of calcium-binding proteins. In addition, HET cells had a deficiency of IVL and TGM1, 2 key regulators of the cornified cell envelope, a highly cross-linked protein meshwork assembled at the inner surface of differentiated keratinocytes that makes the outermost epidermal layers water-impermeable (29, 43). The *TGM1* gene, in particular, has been linked to defective keratinocyte differentiation in patients with ARCI (46).

While many desmosome-associated proteins, including desmogleins (DSG2, DSG3), JUP, DSP, and plakophilins (PKP2, PKP3), exhibited little change in SERCA2-deficient cells, their primary associated intermediate filament cytoskeletal components in the suprabasal epidermal layers, KRT1 and KRT10, were depleted. This finding was verified by immunoblotting HET and KO lysates, which exhibited reduced KRT10, while KRT14 was not notably altered (Figure 3B). If desmosomal complexes are not coupled to the keratin cytoskeleton, this dramatically compromises their ability to support intercellular adhesive strength (33). Consistent with their impaired cohesion in the mechanical dissociation assay, SERCA2-deficient keratinocytes displayed severely attenuated localization of desmosomal proteins to intercellular junctions compared with control cells (Supplemental Figure 2).

Informed by our proteomics data, we aimed to determine if these alterations in SERCA2-deficient keratinocytes translated into pathogenic effects within a human tissue model. We grew KO and HET cells as organotypic skin cultures, which replicate the 3-dimensional architecture of the stratified epidermis (22). Histologic analysis of epidermal cultures of HET cells revealed widening of intercellular spaces and marked disorganization of the upper keratinocyte layers undergoing the final stage of differentiation (Figure 3C). Cornifying cells exhibited cytoplasmic vacuolization, impaired flattening, and retention of nuclei in the outermost keratinized layers (Figure 3C, insets). These histologic defects occurred to a more extreme degree in KO cultures. While direct assessment of intercellular adhesive strength showed markedly reduced cohesion of SERCA2-deficient keratinocyte sheets (Figure 1, C and D), organotypic cultures of these cells did not demonstrate full splitting between keratinocytes (acantholysis), which may reflect differences in shear forces on the in vitro tissue model versus those experienced by the epidermis of patients, who exhibit trauma-induced blistering in areas of friction (10).

Consistent with our proteomics data, immunostaining SERCA2-deficient organotypic epidermis showed reduced KRT10 in the suprabasal layers, which exhibited patchy expression of the cytoskeletal protein compared with the more uniform pattern seen in control cultures (Figure 3, D and E, upper panels). Since KRT10 is a major regulator of epidermal integrity and differentiation (47–49), a concomitant reduction of KRT10 in SERCA2-depleted epidermis could be responsible for the impaired intercellular adhesion and abnormal cornification seen in HET organotypic epidermis, which overlap with the pathologic features seen in DD biopsies.

We and others have previously shown that KRT10 expression is disrupted upon epidermal hyperactivation of MEK and ERK in the MAPK pathway (37, 50, 51). In fact, rises in cytoplasmic calcium, known to drive keratinocyte differentiation (27, 52), have been shown to trigger mitogen-independent MAPK signaling, which can be augmented by SERCA2 blockade (53). Further connecting this signaling pathway to DD, a DD-like disorder called Grover disease, characterized by similar defects in epidermal adhesion and keratinocyte cornification, has been linked to aberrant activation of MEK in patients (54, 55). These observations coupled with our transcriptomics and proteomics analyses led us to hypothesize that SERCA2 deficiency leads to aberrant activation of the MAPK pathway, which could represent a druggable target for treating DD (56).

Thus, we assessed whether hyperactivation of MEK, which functions in the MAPK pathway by activating ERK via phosphorylation, could explain the defects in adhesion and differentiation seen in our DD tissue model. In agreement with this hypothesis, SERCA2-deficient keratinocytes displayed prolonged ERK activation compared with control cells following short-term exposure to increased extracellular calcium (Supplemental Figure 3A). Reflecting longer term MEK hyperactivation in SERCA2-deficient keratinocytes, we found by immunostaining that p-ERK was significantly elevated in confluent monolayers and mature organotypic epidermal cultures of HET cells compared with controls (Figure 3, D and E, lower panels; and Supplemental Figure 3B). In both monolayer and organotypic cultures, we found that p-ERK levels were inversely correlated with the expression of KRT10 (Figure 3, D and E, upper panels; and Supplemental Figure 3C). These findings led us to examine whether suppressing MEK overactivation could restore adhesive protein expression and rescue the phenotype of SERCA2 loss of function.

### Chemical inhibition of SERCA2 in keratinocytes and organotypic epidermis replicates features of DD pathology and induces ERK activation.

**Figure 4:**
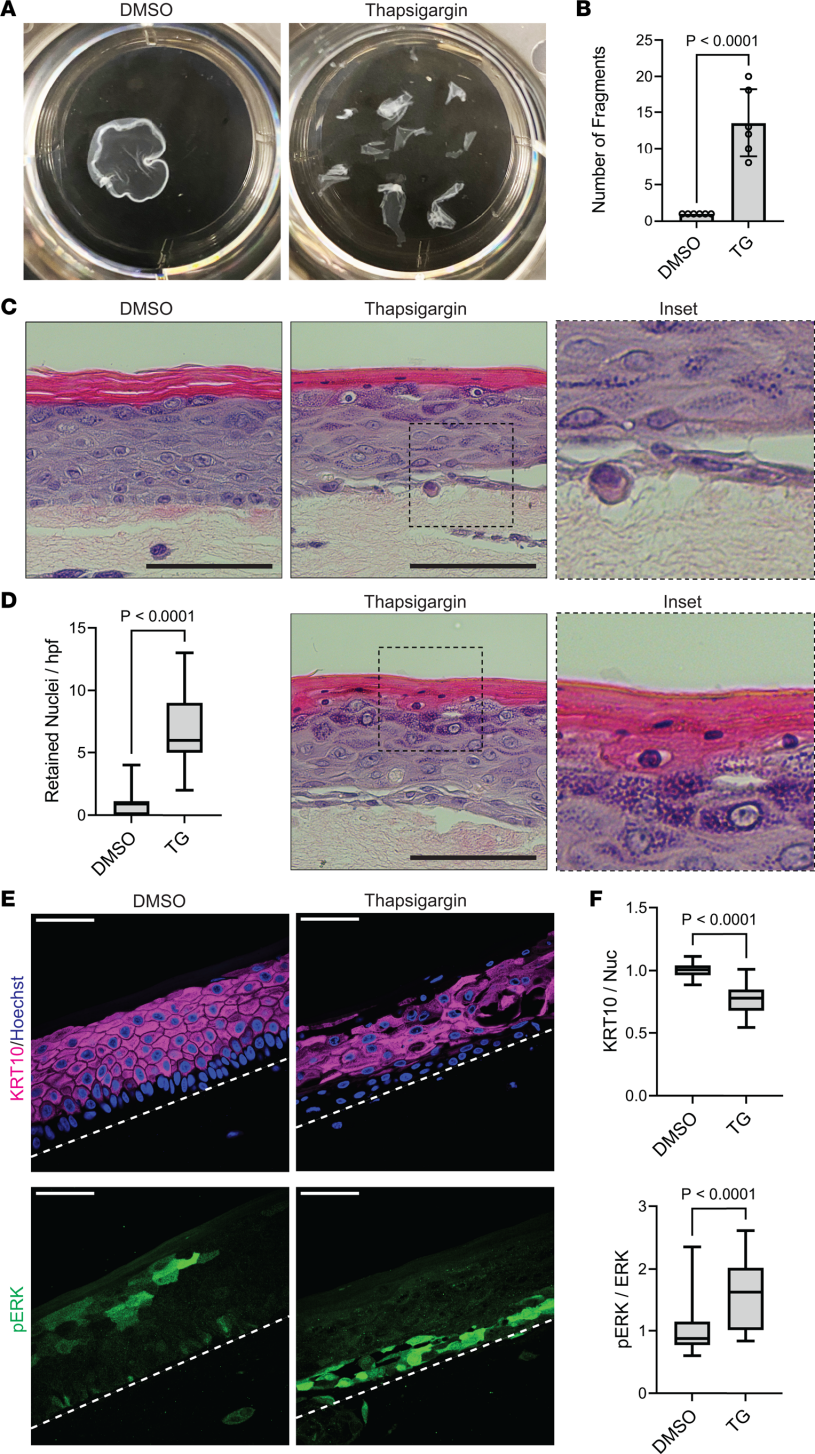
Chemical inhibition of SERCA2 in keratinocytes and organotypic epidermis replicates features of DD pathology and induces ERK activation. (**A**) Mechanical dissociation of confluent monolayers from THEKs cultured in 1.3 mM CaCl_2_ with DMSO or 1 μM thapsigargin (TG) for 24 hours; representative images of fragmented monolayers transferred into 6-well cell culture plates are shown. (**B**) Graph displays mean ± SD of the number of fragments with data points for *N* = 6 biological replicates; *P* value from 2-tailed unpaired Student’s *t* test. (**C**) H&E-stained tissue cross sections of organotypic cultures of NHEKs treated for 48 hours with DMSO or 1 μM TG; inset shows separation between basal and suprabasal layers in TG-treated cultures; scale bar = 100 μm. (**D**) Quantification of retained nuclei in cornified layers; data shown as a box plot of the 25th–75th percentile with a line at the median from *N* ≥ 45 nonoverlapping hpf from 3 biological replicates; *P* value from a 2-tailed unpaired Student’s *t* test; (right) H&E-stained tissue cross section from a TG-treated culture shows retention of nuclei in cornified layers (magnified in inset); scale bar = 100 μm; insets, original magnification = 40×. (**E**) Immunostaining KRT10 and p-ERK in tissue cross sections from organotypic cultures of NHEKs after 48 hours of DMSO or TG treatment; scale bar = 50 μm; dashed line marks bottom of epidermis. (**F**) Quantification of tissue immunostaining of KRT10 (relative to Hoechst) and p-ERK (relative to total ERK) in organotypic cultures of NHEKs treated with DMSO or TG; data shown as a box plot of the 25th–75th percentile with a line at the median from *N* ≥ 98 nonoverlapping hpf per condition from 4 biological replicates; control mean normalized to 1; *P* values from 2-tailed unpaired Student’s *t* test.

To complement our results in SERCA2-deficient immortalized keratinocytes, we assessed the effect of a chemical inhibitor of SERCA2, thapsigargin (TG), on normal human epidermal keratinocytes (NHEKs). Treatment of NHEK monolayers with TG caused a significant increase in epithelial sheet fragmentation (Figure 4, A and B), which verified the role of SERCA2 in promoting intercellular adhesion in primary human keratinocytes. Building on these findings, we treated mature organotypic epidermis grown from NHEKs with TG, which disrupted keratinocyte cohesion most notably between basal and suprabasal cell layers (Figure 4C, inset), a pathologic feature of DD (55). Chemical inhibition of SERCA2 also disrupted keratinocyte differentiation, causing retention of nuclei in the cornified layers (Figure 4D, inset), a feature consistent with aberrant epidermal maturation (termed dyskeratosis) seen in DD (55, 57).

Supporting our data from both RNA-Seq and proteomics analysis, TG-treated organotypic epidermis exhibited a reduction in KRT10, with immunostaining of tissue cross sections demonstrating patchy loss of this cytoskeletal element in the suprabasal layers (Figure 4, E and F, upper panels). Disruption of KRT10 function can compromise epidermal integrity and cause abnormal cornification as in patients with *KRT10* mutations in epidermolytic ichthyosis (48, 58, 59). Like in SERCA2-deficient keratinocytes, ERK hyperactivation was also seen upon TG treatment of NHEK epidermal cultures; p-ERK was highest in the lower keratinocyte layers, where intercellular adhesion was most compromised (Figure 4, E and F, lower panels). These findings supported our hypothesis that loss of SERCA2 function disrupted tissue integrity and epidermal differentiation through overactivation of the MAPK pathway via ERK, resulting in depletion of KRT10.

### Biopsies of DD exhibit mislocalization of cadherins, loss of KRT10, and elevated ERK activation.

**Figure 5:**
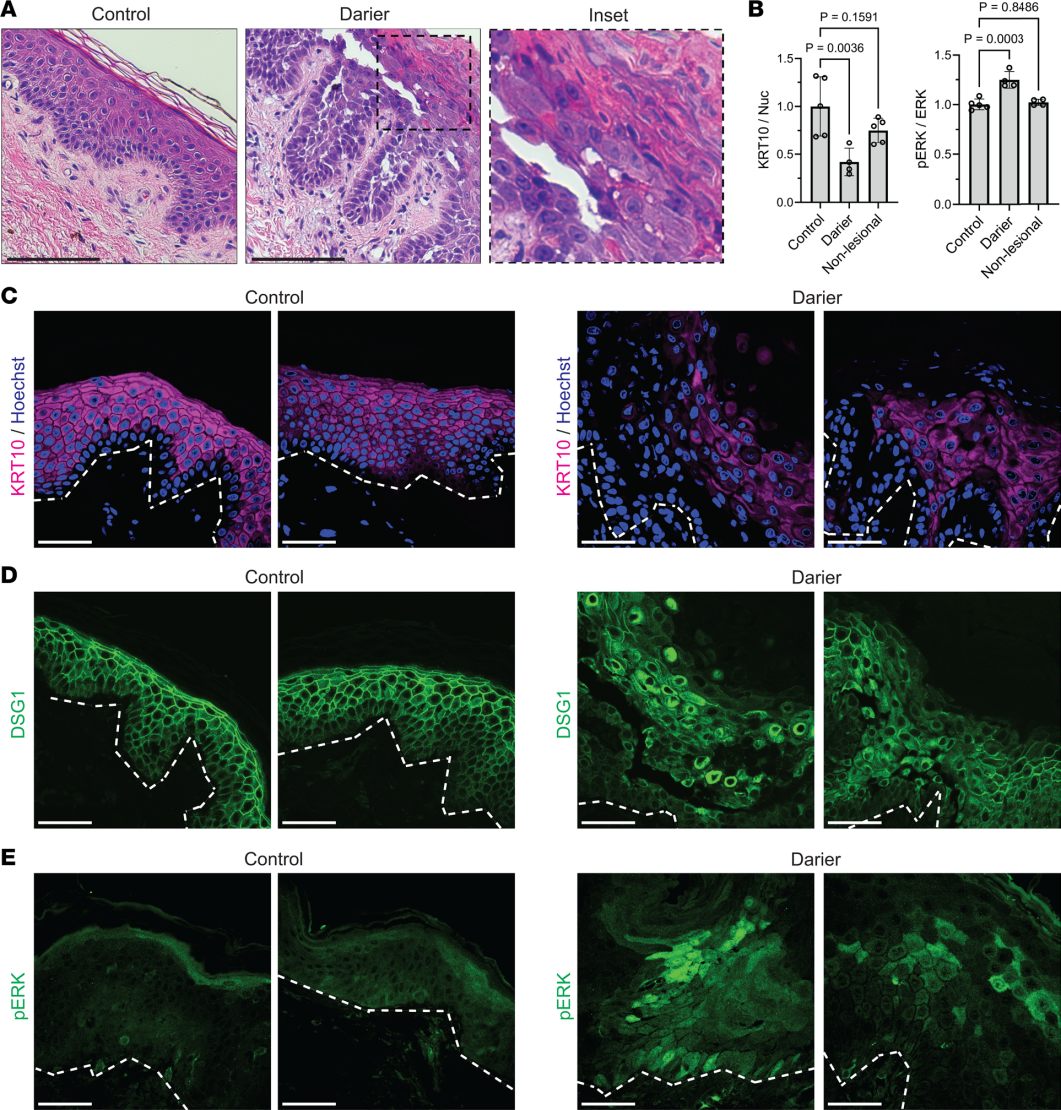
Biopsies of DD exhibit mislocalization of cadherins, loss of keratin 10, and elevated ERK activation. (**A**) H&E-stained cross sections of punch biopsies from skin of control donors versus patients with DD, which demonstrate loss of keratinocyte cohesion (acantholysis) and aberrant cornification (dyskeratosis) with retention of nuclei in cornified layers (magnified in inset; original magnification = 40×); scale bar = 100 μm. (**B**) Quantification of immunostaining of KRT10 (relative to Hoechst) and p-ERK (relative to total ERK) in cross sections of control skin versus in lesional or nonlesional portions of DD biopsies; graph depicts mean ± SD from *N* ≥ 20 nonoverlapping hpf per group from 5 control and 5 DD patients; control mean normalized to 1; *P* values from 1-way ANOVA with Dunnett’s adjustment for multiple comparisons. (**C**) Immunostaining of KRT10, (**D**) DSG1, and (**E**) p-ERK in tissue cross sections from patient biopsies; images from 2 control and 2 DD patients representative of 5 patients in each group; scale bar = 50 μm; dashed line marks bottom of epidermis.

To directly test if our in vitro data from SERCA2-depleted or -inhibited epidermal cultures were reflective of DD pathogenesis, we examined deidentified skin biopsies from patients with DD. Similar to the pathologic features we found in organotypic cultures having SERCA2 genetically ablated or chemically inhibited, histologic examination of DD biopsies demonstrated loss of keratinocyte cohesion and defective differentiation with retention of nuclei in the cornified layers (Figure 5A, inset). Immunostaining biopsies from 5 patients with DD compared with normal control skin demonstrated a significant reduction in KRT10 and a concomitant increase in ERK activation within lesional skin (Figure 5B), consistent with the findings from our in vitro human tissue model. Like in organotypic epidermis, analysis of immunostained DD biopsy cross sections revealed a substantial reduction in overall KRT10 intensity with patchy loss of the cytoskeletal element within DD lesions (Figure 5C).

In addition, we found that protein levels of DSG1, the primary cadherin mediator of desmosomal adhesion in the upper epidermal layers (60, 61), were modestly reduced (data not shown). However, the localization of this cadherin was severely disrupted in DD lesions (Figure 5D), collapsing around the nucleus instead of being concentrated at intercellular junctions. These findings agree with prior in vitro studies showing that keratinocytes having SERCA2 depleted by RNA interference retained normal levels of desmosomal components but exhibited major defects in trafficking of these adhesive building blocks to the plasma membrane, thus impairing the cells’ ability to assemble strong intercellular junctions and compromising monolayer integrity (8, 26, 62, 63).

ERK activation assessed by immunostaining p-ERK, on the other hand, was substantially increased in biopsies from patients with DD compared with biopsies of healthy skin (Figure 5E). These data from DD tissue substantiate our findings from SERCA2-depleted or -inhibited human epidermal cultures and bolster our model in which hyperactivation of the MAPK pathway via ERK is a pathogenic driver in DD and represents a potential therapeutic target.

### Keratin expression and adhesive integrity in SERCA2-deficient keratinocytes are rescued by MEK inhibitors.

**Figure 6:**
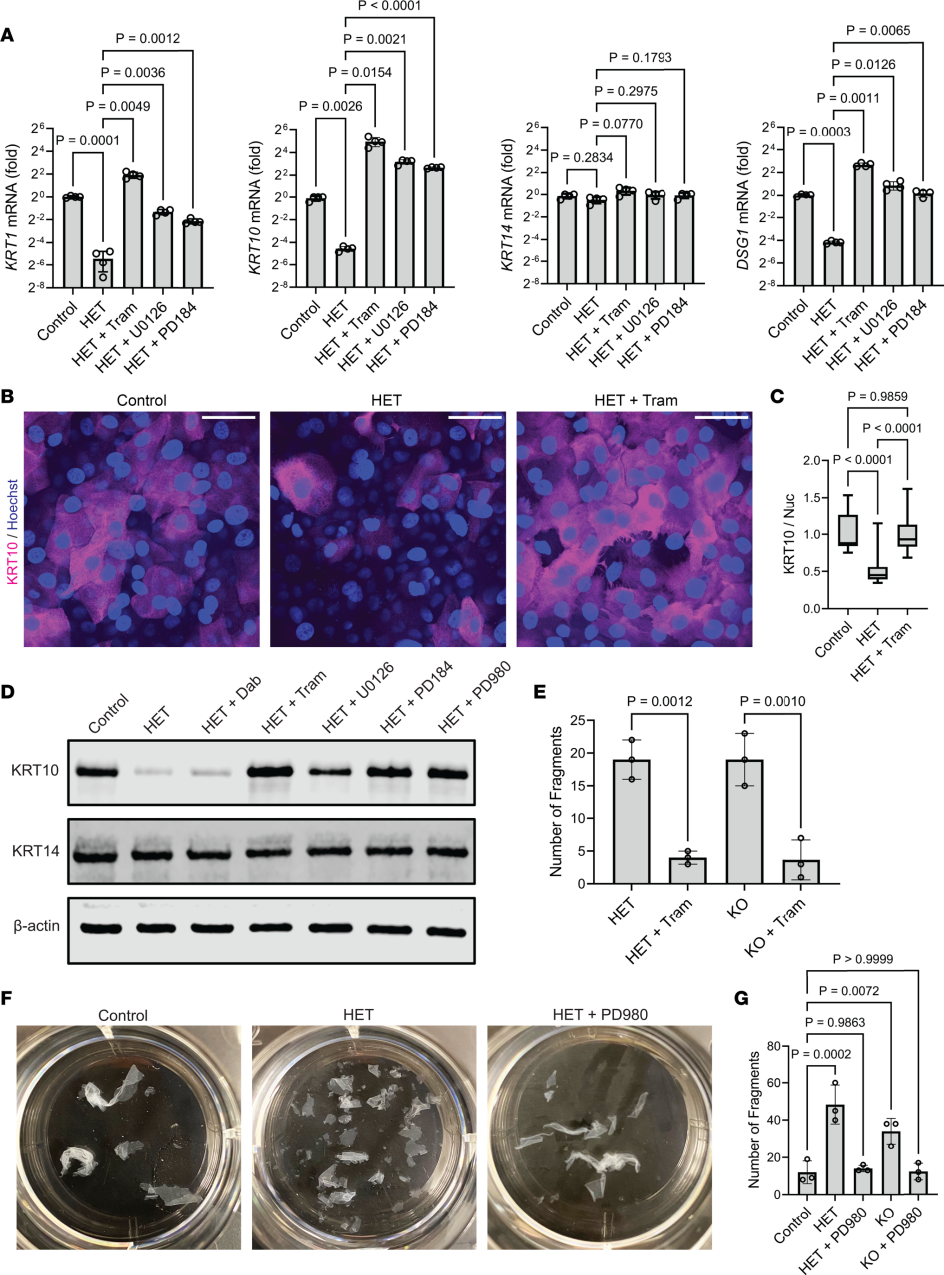
Keratin expression and adhesive integrity in SERCA2-deficient keratinocytes are rescued by MEK inhibitors. (**A**) Control or HET cells were differentiated in E-medium ± MEK inhibitors for 24 hours, and then transcripts were measured by RT-qPCR; graphs show mean ± SD from *N* = 4 replicates; *P* values from ANOVA (Brown-Forsythe) with Dunnett’s T3 adjustment for multiple comparisons. (**B**) Immunostaining KRT10 (and Hoechst) in control or HET cells treated with DMSO or 1 μM trametinib (Tram) for 48 hours; images represent 2 independent experiments using 2 control and 2 SERCA2-deficient lines; scale bar = 50 μm. (**C**) Quantification of KRT10 immunostaining in control versus SERCA2-deficient cells treated with DMSO or 1 μM Tram for 24 hours; data shown as a box plot of 25th–75th percentile with line at the median from *N* ≥ 21 nonoverlapping hpf per group; control mean normalized to 1; *P* values from 1-way ANOVA with Tukey’s adjustment for multiple comparisons. (**D**) Immunoblot of KRT10 and KRT14 (β-actin loading control) in lysates from control versus HET cells treated with DMSO, 1 μM dabrafenib (Dab), or MEK inhibitors (1 μM Tram; 10 μM U0126; 10 μM PD184; 20 μM PD980); data represent 2 independent experiments. (**E**) Quantification of fragments of HET and KO monolayers treated with DMSO or 1 μM trametinib for 48 hours; graphs display mean ± SD for *N* = 3 replicates; *P* values from 1-way ANOVA with Tukey’s adjustment for multiple comparisons. (**F**) Images of 6-well culture plates containing fragmented monolayers of control or HET cells treated with DMSO or 20 μM PD980 for 24 hours. (**G**) Quantification of fragments of control, HET, or KO monolayers treated with DMSO or 20 μM PD980 for 24 hours; graphs display mean ± SD for *N* = 3 replicates; *P* values from 1-way ANOVA with Dunnett’s adjustment for multiple comparisons.

We next assessed the effect of multiple selective MEK inhibitors on SERCA2-depleted keratinocytes to determine if elevated MEK activity directly contributes to the phenotype of SERCA2 deficiency. Quantification of keratinocyte mRNA levels by RT-qPCR again verified that HET cells exhibited significantly reduced expression of the suprabasal keratins, KRT1 and KRT10, and DSG1 while the mRNA encoding KRT14 was not significantly different from controls (Figure 6A). Importantly, treatment of HET cells with any of 3 MEK inhibitors (trametinib, U0126, PD184352) was sufficient to augment expression of KRT1, KRT10, and DSG1, while they did not significantly alter KRT14 mRNA levels.

These differences in mRNA translated to changes in protein levels, with HET cells exhibiting significantly reduced KRT10 as shown by immunostaining differentiated keratinocyte cultures (Figure 6B). Consistent with its effect on *Krt10* mRNA, MEK inhibition increased KRT10 protein, making its levels comparable to those of control cells (Figure 6C). Assessing keratinocyte lysates by immunoblot similarly demonstrated a reduction in KRT10, which multiple individual MEK inhibitors restored to near the baseline level of controls (Figure 6D). In contrast, KRT14 levels were comparable in control and HET cells and were not appreciably altered by MEK inhibitors. Interestingly, targeting the MAPK pathway upstream of MEK using dabrafenib to inhibit RAF failed to rescue KRT10 expression. This result is consistent with prior data indicating that chronic suppression of RAF leads to paradoxical downstream *elevation* in MEK activity (64). This unintended result of RAF blockade changed the standard of care for patients with RAF-driven cancers to include dual RAF and MEK inhibitors to mitigate adverse effects of RAF inhibitors (54, 65). In fact, MEK hyperactivation in patients treated with RAF inhibitor monotherapy can cause a DD-like skin eruption, with biopsies showing impaired keratinocyte adhesion and defective epidermal cornification (66–69).

Since trametinib robustly rescued the expression of adhesive proteins in HET cells and is an FDA-approved drug for the treatment of RAF-mutated cancers (70, 71), we investigated whether this compound could augment intercellular adhesion in SERCA2-deficient keratinocytes. Indeed, treatment with trametinib enhanced the resistance of HET or KO keratinocyte sheets to disruption by mechanical stress (Figure 6E). An additional MEK inhibitor, PD98059, similarly rescued cohesion of SERCA2-depleted cultures (Figure 6, F and G), further supporting the potential clinical utility of inhibiting this kinase to treat DD.

### MEK inhibitors promote keratinocyte cohesion and mitigate epidermal tissue disruption from SERCA2 inhibition.

**Figure 7:**
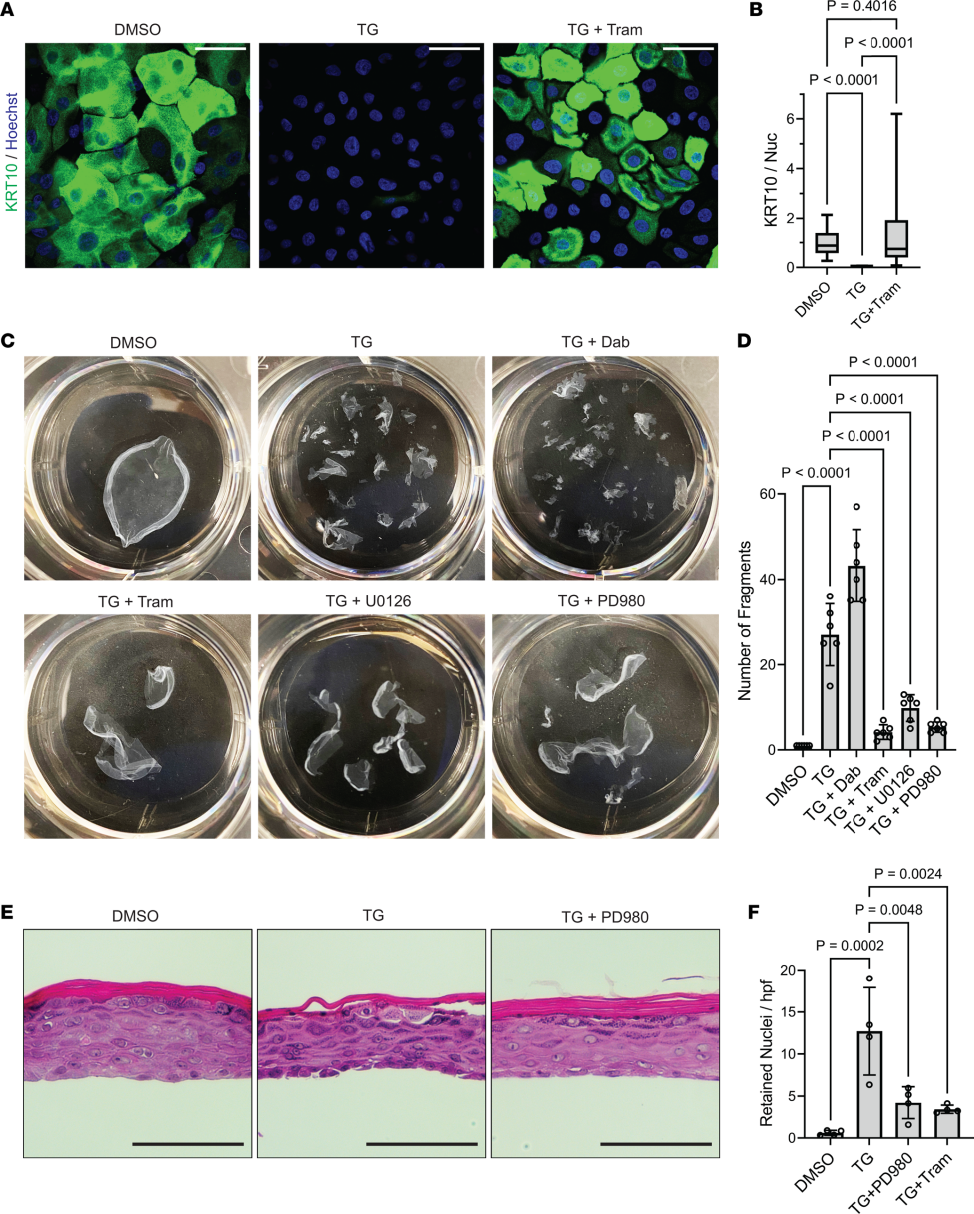
MEK inhibitors promote keratinocyte cohesion and mitigate epidermal tissue disruption from SERCA2 inhibition. (**A**) Immunostaining of KRT10 (and Hoechst) in THEKs treated with DMSO, 1 μM TG, or 1 μM TG plus 1 μM Tram (TG + Tram) for 48 hours; scale bar = 50 μm. (**B**) Quantification of KRT10 immunostaining in THEKs treated with DMSO, TG, or TG plus Tram; data shown as a box plot of the 25th–75th percentile with a line at the median from *N* ≥ 31 nonoverlapping hpf from 2 independent THEK lines for both control and SERCA2-deficient cells; control mean normalized to 1; *P* values from 1-way ANOVA with Tukey’s adjustment for multiple comparisons. (**C**) Representative images of fragmented monolayers transferred into 6-well cell culture plates are shown for NHEKs treated with DMSO versus 1 μM TG alone or with 1 μM dabrafenib versus an MEK inhibitor (1 μM Tram, 10 μM U0126, or 20 μM PD980) for 24 hours. (**D**) Quantification of epithelial fragments of NHEK monolayers; bar graphs display mean ± SD with data points for *N* = 6 biological replicates; *P* values from 1-way ANOVA with Tukey’s adjustment for multiple comparisons. (**E**) H&E-stained tissue cross sections of organotypic cultures of NHEKs treated with DMSO, 1 μM TG, or 1 μM TG plus 20 μM PD980, the latter displaying improved keratinocyte cohesion and normalization of cornification; scale bar = 100 μm. (**F**) Quantification of retained nuclei in cornified layers of organotypic cultures treated with the indicated inhibitors for 48 hours; graph displays mean ± SD with plotted values averaged from ≥49 nonoverlapping hpf per condition from *N* = 4 biological replicates; *P* values from 1-way ANOVA with Tukey’s adjustment for multiple comparisons.

To verify that ERK hyperactivation drives the pathogenic effects of reduced SERCA2 function, we also tested whether MEK inhibitors could reverse TG-induced loss of keratinocyte adhesion and impaired epidermal differentiation. Treatment of NHEKs with TG suppressed their ability to upregulate KRT10 as indicated by immunostaining differentiated keratinocytes (Figure 7, A and B), and this effect of TG was mitigated by concomitant treatment with trametinib to inhibit MEK.

This effect of MEK suppression on KRT10 in SERCA2-inhibited keratinocytes translated into a restoration of intercellular adhesive strength. While TG-treated NHEK monolayers readily fragmented upon mechanical stress, the integrity of epithelial sheets was restored by MEK inhibition with trametinib, U0126, or PD98059 despite SERCA2 inhibition (Figure 7, C and D). In contrast, upstream inhibition of RAF with dabrafenib did not promote intercellular adhesion, but rather exacerbated the effect of TG, further reducing the integrity of NHEK monolayers. These results are consistent with the reported ability of dabrafenib monotherapy to cause paradoxical hyperactivation of MEK (64), which we propose drives the pathogenic effects of SERCA2 deficiency and would explain the similar findings in biopsies of RAF inhibitor–induced Grover disease and DD.

We next treated mature organotypic epidermis with TG either alone or with PD98059 or trametinib to determine if MEK inhibition could prevent DD-like pathologic changes in organotypic epidermis. While TG-treated cultures exhibited loss of keratinocyte cohesion within the epidermal tissue, this effect was mitigated by MEK inhibitors (Figure 7E). Moreover, treatment with PD98059 or trametinib reduced TG-induced retention of nuclei in the cornified layers (Figure 7F), indicating that MEK inhibition could normalize epidermal differentiation in DD. Together, these data support our model in which the pathogenic effects of SERCA2 loss of function in epidermis are driven by ERK hyperactivation, which can be mitigated by MEK inhibition.

## Discussion

The advent of rational molecular therapeutics has revolutionized the treatment of many dermatologic conditions (4, 5); however, most advances have been made for common inflammatory diseases, like psoriasis and atopic dermatitis, driven by soluble cytokines amenable to targeting by monoclonal antibodies (72, 73). Unfortunately, skin blistering diseases and disorders of cornification are rare and most lack any FDA-approved treatments; although recent work suggests cytokine inhibition may improve DD and certain types of ichthyosis, these medications target inflammatory pathways, which are likely downstream of defective skin barrier function, rather than targeting the primary dysfunction of keratinocytes themselves (74–79). While we did not identify an inflammatory signature in our keratinocyte cultures, SERCA2-deficient cells had reduced IFI27 and other interferon response genes, which might explain the susceptibility of DD lesions to viral infection (13). For DD, the knowledge of its genetic etiology has not significantly advanced the treatment of this chronic, incurable disorder. Though retinoids, which modulate keratinocyte differentiation, may be helpful in DD (14, 15), they are potent teratogens and can cause multisystem toxicity with long-term use (17, 18). Some evidence suggests reducing ER stress may be a viable alternative approach for DD treatment (9), though this strategy has not yet been proved clinically.

Using mice as a preclinical model has enabled important discoveries about disease pathogenesis that translated into new therapies for patients, but animal models do not always phenocopy human pathology, as with disrupting *Atp2a2* in mice (20, 21). The development of organoid models to replicate human tissues in vitro has the potential to revolutionize the way we investigate disease pathogenesis, identify lead compounds, and screen for therapeutic efficacy and toxicity (80, 81). Here, we leveraged gene editing in human keratinocytes and an organotypic epidermal model to elucidate the pathogenic mechanisms underlying a rare genetic disorder, which allowed us to identify much-needed potential therapeutic avenues. Though testing if our findings translate to an effective treatment approach for patients remains to be done, our work nevertheless underscores the power of organoids to generate large-scale transcriptomics and proteomics data sets that can be mined for rational drug targets to validate both in vitro and in patient-derived tissues (80, 82–85).

While the primary DD pathology derives from impaired ER calcium homeostasis (26, 57), direct targeting of SERCA2 is likely to be therapeutically challenging. Recent intravital imaging of murine epidermis revealed that calcium flux undergoes dramatic shifts during epidermal proliferation (86) and in the late stages of cornification (30). Thus, sustained drug-induced activation of SERCA2 could compromise the dynamic nature of calcium-mediated signals critical for epidermal functions (27, 28). Since calcium is a major second messenger regulating diverse biological functions, including skeletal muscle contraction, cardiac pacing, neurotransmitter release, and apoptosis in many cell types (87, 88), compounds affecting intracellular calcium may have a narrow therapeutic window. In fact, coincidence of neuropsychiatric and cardiac disease with DD (6, 89) implies a role beyond the skin for SERCA2 (90), including in higher order cognition and behavior (91–93).

The study of rare diseases can yield unexpected findings that improve our understanding of fundamental biology or the pathology of more common disorders (94). While our study focused on DD, our results demonstrating ERK hyperactivation in DD bring to mind a noninherited dermatologic disorder called Grover disease, which can be induced by RAF inhibition (66, 67, 69). Given that biopsy findings in Grover disease can be indistinguishable from DD (55), it seems likely that they share a common pathogenic mechanism. Indeed, recent work demonstrated that a subset of Grover disease cases arise from damaging somatic mutations in the *ATP2A2* gene (95). Finally, the fact that RAF inhibitor–induced Grover disease is suppressed by adding a MEK inhibitor further supports our hypothesis that MEK inhibitors would be effective for DD (54, 65).

In sum, our findings indicate that excess ERK signaling disrupts the differentiation and cohesion of epidermal keratinocytes (37, 39, 40, 51) to drive the pathogenesis of DD, which can be reversed by inhibiting MAPK signaling through MEK. Whether this pathway could be similarly targeted for therapeutic benefit in other diseases compromising desmosomal adhesion remains to be tested, but extensive data previously implicated a pathogenic role for the MAPK pathway in pemphigus via p38 (96–98), which unfortunately did not progress to clinical approval (99). However, given that multiple MEK inhibitors are already approved for clinical use with long-term data supporting their safety (56, 65, 70, 100), we propose that MEK inhibition could represent a viable therapeutic strategy for both inherited and acquired blistering diseases and disorders of cornification, all of which are in great need of novel treatments.

## Methods

### Reagents.

The SERCA2 inhibitor thapsigargin (catalog 12758); MEK inhibitors trametinib (catalog 62206), U0126 (catalog 9903), PD98059 (catalog 9900), and PD184352 (catalog 12147); and B-Raf inhibitor dabrafenib (catalog 91942) are from Cell Signaling Technology. Rabbit antibodies against SERCA2 (D51B11; catalog 9580) and p-ERK1/2 (D13.14.4E; catalog 4370) as well as mouse antibodies against ERK1/2 (L34F12; catalog 4696) and β-actin (8H10D10; catalog 3700) are from Cell Signaling Technology. Rabbit anti–Cytokeratin 10 (catalog ab76318), mouse anti–Cytokeratin 14 (catalog ab7800), and mouse anti–Desmoglein 1 (catalog ab12077) are from Abcam. Mouse anti–Desmoglein 2 (AH12.2; catalog sc-80663), mouse anti–Desmoglein 3 (5G11; catalog sc-53487), mouse anti–β-actin (C4; catalog sc-47778), and mouse anti-Plakoglobin (A-6; catalog sc-514115) are from Santa Cruz Biotechnology. Mouse anti-SERCA2 (IID8; catalog MAB2636) is from MilliporeSigma. For fluorescence immunoblotting, IRDye 800CW goat anti-rabbit IgG (catalog 926-32211) and IRDye 680RD goat anti-mouse IgG (catalog 926-68070) are from LI-COR Biosciences. For fluorescence immunostaining, secondary antibodies are from Thermo Fisher Scientific: goat anti-mouse IgG with Alexa Fluor 405 (catalog A31553), Alexa Fluor 488 (catalog A11001), Alexa Fluor 594 (catalog A11005), or Alexa Fluor 633 (catalog A21050) and goat anti-rabbit IgG with Alexa Fluor 405 (catalog A31556), Alexa Fluor 488 (catalog A11008), Alexa Fluor 594 (catalog A11012), or Alexa Fluor 633 (catalog A21070). Hoechst 33342 is from Thermo Fisher Scientific (catalog H1399).

### Cell culture.

THEKs from original line N/TERT-2G (31) (a gift from James Rheinwald, Brigham and Women’s Hospital, Boston, Massachusetts, USA) were grown in keratinocyte serum-free medium (KSFM) ordered as a kit (Thermo Fisher Scientific catalog 37010022) with supplements of 0.2 ng/mL human epidermal growth factor and 30 μg/mL bovine pituitary extract. Other additives included 0.31 mM CaCl_2_, 100 U/mL penicillin, and 100 μg/mL streptomycin.

NHEKs were procured as described below and grown in Medium 154 with 0.07 mM CaCl_2_ (Thermo Fisher Scientific catalog M154CF500), 1× human keratinocyte growth supplement (Thermo Fisher Scientific catalog S0015), and 1× gentamicin/amphotericin (Thermo Fisher Scientific catalog R01510).

J2-3T3 immortalized murine fibroblasts (a gift from Kathleen Green, Northwestern University, Chicago, Illinois, USA) were grown in complete DMEM (Thermo Fisher Scientific catalog 11965092) supplemented with 10% FBS (Hyclone, Thermo Fisher Scientific catalog SH3039603), 2 mM GlutaMAX (Thermo Fisher Scientific catalog 35050061), 100 U/mL penicillin, and 100 μg/mL streptomycin.

All cell lines were maintained at 37°C in 5% CO_2_ in an air-jacketed, humidified incubator. Cells were grown on sterile cell culture dishes and passaged at subconfluence using 0.25% Trypsin-EDTA (Thermo Fisher Scientific catalog 15400054).

### Organotypic epidermal culture.

Human organotypic epidermal “raft cultures” were generated as described (22, 101). Cultures were differentiated using E-medium, a 3:1 mixture of DMEM/Ham’s F12 (Thermo Fisher Scientific catalog 11765054) with 10% FBS, 180 μM adenine (MilliporeSigma catalog A2786), 0.4 μg/mL hydrocortisone (MilliporeSigma, catalog H0888), 5 μg/mL human insulin (MilliporeSigma catalog 91077C), 0.1 nM cholera toxin (MilliporeSigma, catalog C8052), 5 μg/mL apo-transferrin (MilliporeSigma catalog T1147), and 1.36 ng/mL 3,3′,5-tri-iodo-l-thyronine (MilliporeSigma catalog T6397).

J2-3T3 fibroblasts were seeded into collagen matrix rafts within Transwells (Corning catalog 353091). For each raft, 1 × 10^6^ fibroblasts were resuspended in 1/10 the final volume of sterile filtered reconstitution buffer (1.1 g of NaHCO_3_ plus 2.39 g of HEPES in 50 mL 0.05N NaOH), and then 1/10 the final volume of 10× DMEM (MilliporeSigma catalog D2429) was added. The cells were mixed thoroughly by pipetting, and then high-concentration rat tail collagen I (Corning catalog CB354249) was added (4 mg/mL final concentration), along with sterile deionized H_2_O to bring the solution up to the final volume. If necessary, 0.05N NaOH was added dropwise to adjust the pH to approximately 7 based on the phenol red indicator. The collagen-fibroblast slurry was mixed via inversion, then pipetted into a Transwell insert placed within deep, 6-well cell culture plates (Corning catalog 08-774-183). The rafts were polymerized at 37°C for 1 hour, after which they were submerged in complete DMEM and incubated at 37°C overnight.

Next, confluent keratinocyte cultures were trypsinized and resuspended in E-medium with EGF (5 ng/mL) to final concentration of 0.5 × 10^6^ cells/mL (2 mL per organotypic culture). The DMEM was aspirated from the upper and lower Transwell chambers, and then 2 mL (1 × 10^6^ cells) of the keratinocyte suspension were pipetted on top each raft. E-medium with EGF was added to the bottom Transwell chamber to submerge the raft, and the cultures were incubated at 37°C. After 24 hours, E-medium was aspirated from the top and bottom chambers. An air-liquid interface was established to induce stratification by adding E-medium (without EGF) only to the bottom chamber to reach the bottom of the raft. Organotypic cultures were grown at 37°C for up to 12 days. E-medium in the bottom chamber was replaced every other day. For drug treatments, inhibitor or vehicle control (DMSO) was diluted in E-medium in the bottom chamber. Inhibitors were used at the following concentrations: thapsigargin (1 μM), trametinib (1 μM), and PD98059 (20 μM). For histologic examination, the Transwell was moved to a standard 6-well cell culture plate and submerged in 10% neutral-buffered formalin (Thermo Fisher Scientific catalog 22-026-435) for at least 24 hours. Organotypic cultures were processed for histologic examination by Core A of the Penn Skin Biology and Diseases Resource-based Center (SBDRC) or the Experimental Histopathology Core of the Fred Hutchinson Cancer Center.

### CRISPR/Cas9 gene editing.

CRISPR-KO keratinocytes were generated as described (24). Single-guide RNAs (sgRNAs) were designed to target the *ATP2A2* gene (sgRNA2: GTTTTGGCTTGGTTTGAAGA) or the *TUBAP* pseudogene (sgRNA1: GTATTCCGTGGGTGAACGGG) to generate a control KO line using a web tool for CRISPR design (https://portals.broadinstitute.org/gpp/public/analysis-tools/sgrna-design). Synthetic sgRNA target sequences were inserted into a cloning backbone, pSpCas9 (BB)-2A-GFP (PX458) (Addgene, catalog 48138), then transformed into competent *E*. *coli* (Thermo Fisher Scientific, catalog C737303). Proper insertion was validated by Sanger sequencing. The final plasmid was transfected into an N/TERT-2G line (31) using the TransfeX transfection kit (ATCC catalog ACS4005) in the presence or absence of JAK1/JAK2 inhibitor baricitinib (10 μg/mL). GFP^+^ single cells were plated and expanded. Cells were genotyped and analyzed by Sanger sequencing to confirm the presence of heterozygous or homozygous mutations in the target gene.

### Determination of cell proliferation.

THEKs were seeded at a density of 1.25 × 10^5^ cells per well of 6-well culture dishes in complete KSFM. Cells were then transferred to a CELLCYTE X live-cell imaging system to determine proliferation rates of label-free cells. Images of 25 nonoverlapping fields per well were acquired every 2 hours using the 10× objective in the enhanced contour channel. Images were analyzed using the CELLCYTE analysis software to measure cell confluence, and growth rates were extrapolated by simple linear regression using GraphPad Prism software.

### RNA-Seq.

RNA-Seq libraries were prepared for transcriptomics analysis as described (102). THEKs were grown to confluence in 10 cm cell culture dishes in KSFM, then differentiated in E-medium for 72 hours. RNA was isolated from THEKs using the RNeasy Kit (QIAGEN) according to the manufacturer’s instructions. mRNAs were isolated using the NEBNext Poly(A) mRNA magnetic isolation module (New England Biolabs). RNA-Seq libraries were prepared for Illumina using the NEBNext Ultra-Directional RNA library preparation kit (New England Biolabs). Library quality was confirmed using an Agilent BioAnalyzer 2100 and quantified using the NEBNext Library Quant Kit for Illumina (New England Biolabs). Sequencing was performed using the Illumina NextSeq500 platform employing a single-end, 75-base pair sequencing strategy. All RNA-Seq reads were then aligned to the *Homo sapiens* reference genome (UCSC hg19, RefSeq and Gencode gene annotations) using RNA STAR under default settings. Fragments per kilobase per million mapped fragments generation and differential expression analysis were done using the DESeq2 package. Statistical significance was determined using an adjusted *P* value ≤ 0.05.

### GO analyses.

GO analyses of RNA-Seq data were performed as described (102) using PANTHER (http://pantherdb.org/). All human genes in the database were used as the reference list. Enrichment analysis under the category “biological process” was performed to identify statistically overrepresented GO terms. Statistical analysis was performed in PANTHER using Fisher’s exact test and FDRs were calculated by the Benjamini-Hochberg procedure. Normalized enrichment scores and FDRs were computed for each biological process. GO terms were plotted using Prism 9.

### Whole-cell proteomics.

THEKs were grown to confluence in 10 cm cell culture dishes in complete KSFM, then differentiated in E-medium for 72 hours. Cells were then washed with PBS and lysed in ice-cold lysis buffer (0.1 M ammonium bicarbonate, 8 M urea, 0.1% [w/v] RapiGest SF from Waters). Lysates were homogenized using a microtip probe sonicator (Thermo Fisher Scientific) and clarified by centrifugation at 10,000*g* for 5 minutes at 4°C. Clarified supernatants were transferred to a new microcentrifuge tube, and protein concentrations were determined by BCA assay (Pierce). After normalizing sample volumes, 200 μg of protein from each sample was reduced by the addition of tris carboxy ethyl phosphene to a final concentration of 5 mM for 1 hour at room temperature. Protein samples were then alkylated for 30 minutes at room temperature by adding iodoacetamide to a final concentration of 10 mM. Unreacted iodoacetamide was quenched by adding TCEP to a final concentration of 10 mM for 30 minutes at room temperature. Following reduction and alkylation, samples were diluted with 0.1 M ammonium bicarbonate to decrease the urea concentration to 1.5 M. Protein samples were then digested overnight at 37°C with Trypsin Gold (Promega) according to the manufacturer’s instructions. On the following day, each sample was adjusted to pH 2 with 2 M HCl to hydrolyze the RapiGest surfactant. The precipitated RapiGest was then removed via centrifugation at 10,000*g* for 5 minutes at 4°C. Acetonitrile and trifluoroacetic acid were added to the clarified supernatants to final concentrations of 5% (v/v) and 0.1% (v/v), respectively. Trypsin-digested peptides were then desalted using MacroSpin C18 columns (Pierce), according to the manufacturer’s instructions. Following elution, peptides were completely dried under vacuum and resuspended in 0.1% (v/v) formic acid to a final concentration of approximately 0.5 μg/mL. Samples were transferred into autosampler vials and subjected to MS analysis using an Orbitrap Eclipse mass spectrometer (Thermo Fisher Scientific) as described (103). Raw spectral data were processed in MaxQuant using the *Homo sapiens* reference protein library (UniProt), and relative protein abundances were determined using the LFQ intensity method as described (103). Statistical analysis was performed using a 2-tailed unpaired Student’s *t* test.

### RNA isolation and real-time quantitative PCR.

THEKs were seeded at a density of 1 × 10^6^ cells per well of 6-well cell culture dishes in complete KSFM. Upon reaching confluence, KSFM was replaced with E-medium containing vehicle control (DMSO) or inhibitors at the following concentrations: trametinib (1 μM), U0126 (10 μM), or PD184352 (10 μM). At the indicated time points, cell culture medium was removed, and cells were washed once with PBS. RNA was extracted using the NucleoSpin RNA Plus Kit (Macherey-Nagel) followed by reverse transcription with the iScript cDNA synthesis kit (Bio-Rad) according to the manufacturer’s instructions. cDNAs were diluted 5-fold and 2 μL of the resulting cDNAs were used for each 10 μL qPCR assay. Real-time quantitative PCR assays were carried out using the TaqMan Gene expression master mix (Applied Biosystems) according to the manufacturer’s protocol. Predesigned TaqMan probes from Thermo Fisher Scientific were used for quantification of human *ACTB* (Hs01060665_g1), *KRT1* (Hs00196158_m1), *KRT10* (Hs00166289_m1), *KRT14* (Hs00265033_m1), *DSG1* (Hs00355084_m1), *ALOX12B* (Hs00153961_m1), *ALOX15B* (Hs00153988_m1), *IFI27* (Hs01086373_g1), *TRPV3* (Hs00376854_m1), *TRPV4* (Hs01099348_m1), or *NOTCH1* (Hs01062014_m1). The housekeeping gene *ACTB* was used as an endogenous control, and mRNA fold-changes were determined using the 2^(-ΔΔCt)^ method.

### Immunoblotting.

THEKs were seeded at a density of 1 × 10^6^ cells per well of 6-well cell culture dishes in KSFM. Upon reaching confluence, KSFM was replaced with E-medium containing vehicle control (DMSO) or inhibitors at the following concentrations: dabrafenib (1 μM), trametinib (1 μM), U0126 (10 μM), PD184352 (10 μM), or PD98059 (20 μM). After 72 hours, whole-cell lysates were generated by washing cells once in PBS followed by lysis in urea sample buffer (8 M urea, 60 mM Tris, 1% SDS, 10% glycerol, 5% β-mercaptoethanol, 0.0005% pyronin-Y, pH 6.8) for 10 minutes. Lysates were homogenized using a microtip probe sonicator (Thermo Fisher Scientific).

Whole-cell lysates were loaded onto Any kD Mini-PROTEAN TGX Precast Protein Gels (Bio-Rad) and separated by electrophoresis. Proteins were transferred onto nitrocellulose membranes (Bio-Rad) in ice-cold Towbin transfer buffer (25 mM Tris, 192 mM glycine, 20% [v/v] methanol) at 100 V for 90 minutes at 4°C. Membranes were blocked in Intercept TBS blocking buffer (LI-COR Biosciences) for 45 minutes at room temperature. Membranes were probed overnight at 4°C with the primary antibodies diluted at 1:1,000 in Intercept TBS blocking buffer. Blots were washed at least 3 times in 1× TBS containing 0.1% (v/v) Tween-20 (TBS-T), then incubated for 1 hour at room temperature with IRDye 800CW goat anti-rabbit IgG and/or IRDye 680RD goat anti-mouse IgG (LI-COR Biosciences) diluted 1:10,000 in Intercept TBS blocking buffer. Blots were washed at least 3 times in TBS-T, and proteins were visualized using an Odyssey Fc Imaging System (LI-COR Biosciences).

### Fluorescence immunocytochemistry.

Keratinocytes were grown to confluence in 35 mm, glass-bottom cell culture dishes (MatTek P35G-1.5-20-C). For staining keratins and desmosomal proteins, cells were fixed in ice-cold 100% methanol at –20°C for 2 minutes, allowed to dry, and then rehydrated in PBS. For staining other proteins, cells were fixed in 4% paraformaldehyde for 10 minutes at 37°C. Fixed cells were then incubated with blocking buffer — 0.5% (w/v) bovine serum albumin (BSA, MilliporeSigma), 10% (w/v) normal goat serum (NGS, MilliporeSigma), in PBS — for 30 minutes at 37°C. Cells were then washed with PBS. Primary antibodies were diluted in 0.5% (w/v) BSA in PBS and incubated on the cells overnight at 4°C. Primary antibody dilutions were as follows: rabbit anti-SERCA2 (1:200), rabbit anti–cytokeratin 10 (1:2,000), mouse anti–Desmoglein 1 (1:100), mouse anti–Desmoglein 2 (1:100), mouse anti–Desmoglein 3 (1:100), mouse anti-Plakoglobin (1:100), rabbit anti–p-ERK1/2 (1:400), and mouse anti-ERK1/2 (1:400). Cells were washed 3 times with PBS, then incubated with species-specific secondary antibodies diluted at 1:300 (with or without Hoechst at 1:500) in 0.5% (w/v) BSA in PBS for 30 minutes at 37°C. Cells were washed 3 times with PBS, then held in PBS for imaging using spinning-disk confocal microscopy, as detailed below.

### Histologic analysis and tissue procurement.

Paraffin-embedded, formalin-fixed tissue cross sections of organotypic epidermis or skin biopsies were processed for histology and stained with H&E using standard methods. H&E-stained glass slides were imaged on an EVOS FL imaging system (Thermo Fisher Scientific) using an EVOS 40×, long–working distance, achromatic, phase-contrast objective (Thermo Fisher Scientific). Images were captured using the embedded high-sensitivity interline CCD color camera.

### Fluorescence immunohistochemistry.

Paraffin-embedded, formalin-fixed tissue cross sections on glass slides were incubated at 65°C for 2 hours. Sections were prepared for staining by immersion in 3 baths of xylene (Fisher Scientific, Thermo Fisher Scientific) for 5 minutes each, followed by 3 baths of 95% ethanol for 5 minutes each, then 70% ethanol for 5 minutes, and finally 3 baths of PBS for 5 minutes each. Slides were then submerged in antigen retrieval solution (0.1 M sodium citrate pH 6.0 with 0.05% v/v Tween-20) and heated to 95°C for 15 minutes. Slides were allowed to cool to room temperature and then washed with PBS. Tissue sections were encircled with a hydrophobic barrier using a PAP pen. Tissue sections were incubated in blocking buffer (0.5% [w/v] BSA, 10% [v/v] NGS in PBS) for 30 minutes at 37°C in a humidified chamber. Slides were washed in 3 baths of PBS for 5 minutes each, then incubated with primary antibodies diluted in 0.5% (w/v) BSA in PBS overnight at 4°C in a humidified chamber. Primary antibody dilutions were as follows: rabbit anti–cytokeratin 10 (1:3,000), mouse anti–desmoglein 1 (1:50), rabbit anti–p-ERK (1:400), and mouse anti-ERK (1:400). Slides were then washed in 3 baths of PBS for 5 minutes each and incubated with secondary antibodies diluted at 1:300 (with or without Hoechst at 1:500) in 0.5% (w/v) BSA in PBS for 60 minutes at 37°C in a humidified chamber. Slides were washed in 3 baths of PBS for 5 minutes each and mounted in Prolong Gold (Invitrogen catalog P36934) with a glass coverslip applied over the tissue sections. Slides were allowed to dry overnight prior to imaging by spinning-disk confocal microscopy, as detailed below.

### Fluorescence microscopy imaging.

Images were acquired on a Hamamatsu ORCA-FusionBT sCMOS camera using a Yokogawa W1 spinning-disk confocal (SDC) system on a Nikon Ti2 microscope. Samples were illuminated using 405, 488, 561, and 640 nm laser excitation lines, and fluorescence was detected using a 60× 1.2 NA water objective (Nikon) with standard emission filters.

For live imaging of cytoplasmic calcium, THEKs were transduced with GCaMP6 (Addgene catalog 40753) subcloned into the pLZRS retroviral vector. HEK293 Phoenix cells were grown in complete DMEM, then transfected with 4 μg pLZRS-GCaMP6 DNA plus 12 μL FuGENE 6 (Promega catalog E2691) in 800 μL of Opti-MEM (Thermo Fisher Scientific catalog 31985070), which was added to the cells and left overnight. Retroviral supernatants were collected the next day, and polybrene (MilliporeSigma catalog H9268) was added at a concentration of 4 μg/mL. KSFM was removed from THEKs and replaced with viral medium for 1 hour at 37°C. Cells were then washed in PBS and placed back in their normal medium and expanded in culture.

GCaMP6-transduced cells were seeded into 35 mm, glass-bottom dishes in low-calcium medium (0.31 mM) and grown to confluence, then were imaged by confocal microscopy at 1 frame every 5 seconds. Cells were exposed to high calcium (1.3 mM) after 60 seconds, then were imaged for an additional 5 minutes at 1 frame every 5 seconds. The Fiji “Measure” function was used to calculate the mean intensity of GCaMP6 signal across the entire hpf of the time series of images. The intensity at 0 seconds was subtracted from the intensity at each subsequent time point to calculate the change from baseline as a function of time across independent replicates for each genotype.

### Fluorescence microscopy quantification.

Fluorescence microscopy images were analyzed using Fiji software. Quantification of fluorescence intensity was performed in a blinded manner using nonvisibly identifiable microscopy images. The Fiji “Measure” function was used to calculate the mean intensity across the entire hpf of fixed cells or a region of interest encompassing the entire epithelium circumscribed using the polygon selection tool. The mean intensity for each condition was averaged across multiple independent hpf for each biological replicate.

### Tissue morphologic quantification.

Counts of retained nuclei in the cornified layers were performed by hand using nonvisibly labeled H&E images to identify and count the numbers of retained nuclei per nonoverlapping hpf for each condition, which were averaged across multiple independent experiments.

### Fluorescence tissue staining quantification.

Fluorescence images of immunostained tissue sections were captured by SDC microscopy as above and analyzed using Fiji. Quantification of fluorescence intensity was performed using nonvisibly labeled images from immunostained tissue cross sections. The Fiji “Measure” function was used to calculate the mean intensity of the fluorescence signal and the mean background fluorescence intensity, which was subtracted from each value. The net fluorescence intensity was averaged across multiple nonoverlapping hpf, and the values from control cultures were normalized to an average value of 1.

### Monolayer mechanical dissociation assay.

Dispase-based mechanical dissociation assays were carried out as described (104). Keratinocytes were plated at a density of 1 × 10^6^ cells per well of 6-well cell culture dishes. Upon reaching confluence, the calcium concentration of the medium was adjusted to 1.3 mM. Vehicle control (DMSO) or chemical inhibitors were added at the following concentrations: thapsigargin (1 μM), dabrafenib (1 μM), trametinib (1 μM), U0126 (10 μM), PD184352 (10 μM), or PD98059 (20 μM). After 24 to 72 hours, monolayers were washed with PBS and then incubated with 500 μL dispase (5 U/mL) in Hank’s balanced salt solution (StemCell Technologies, catalog 07913) for 30 minutes at 37°C. Next, 4.5 mL PBS was added to the wells, and all liquid plus released monolayers were transferred into 15 mL conical tubes, which were placed together in a rack and inverted 5–10 times to induce mechanical stress. Monolayer fragments were transferred back into 6-well cell culture plates and imaged with a 12-megapixel digital camera. Fragments were counted in Fiji.

### Statistics.

Statistical analyses were performed using the open-source statistics package R or Prism version 9 (GraphPad), which was used to generate graphs. Statistical parameters including sample size, definition of center, dispersion measures, and statistical tests are included in each figure legend. Data sets were tested for normality using the D’Agostino-Pearson test. The means of 2 normally distributed groups were compared using a 2-tailed unpaired Student’s t test. The means of more than 2 normally distributed groups were compared using a 1-way ordinary ANOVA followed by *P* value adjustment for multiple comparisons. *P* values less than 0.05 were considered statistically significant. Exact *P* values are included in each figure.

### Study approval.

NHEKs from deidentified neonatal foreskins were procured by the Penn SBDRC under a protocol (808224) approved by the University of Pennsylvania Institutional Review Board (IRB). Tissue cross sections of deidentified skin biopsies were obtained from a tissue bank by the Penn SBDRC under a protocol (808225) approved by the University of Pennsylvania IRB. The use of deidentified tissues collected for clinical purposes that would otherwise be discarded was deemed exempt by the IRB from written informed consent.

### Data availability.

Values from all graphs are available in the Supporting Data Values file. Raw RNA-Seq data and raw proteomics data are also available in the Supporting Data Values file.

## Author contributions

SAZ and CLS conceived the study. SAZ, SE, JZ, AT, BCC, and CLS curated data. SAZ, SE, JZ, AT, BCC, and CLS performed formal analysis. SAZ, MKS, SE, JZ, AT, and CLS performed experiments. BCC, JEG, and CLS provided resources. CLS supervised the study. SAZ, SE, and CLS visualized data. SAZ and CLS validated data. SAZ and CLS wrote the original draft. SAZ, MKS, SE, JZ, AT, BCC, JEG, and CLS reviewed and edited the manuscript.

## Supplementary Material

Supplemental data

Supporting data values

## Version 1 08/10/2023

In-Press Preview

## Version 2 09/22/2023

Electronic publication
