# Supplementary material for: Targeting SERCA2 in organotypic epidermis reveals MEK inhibition as a therapeutic strategy for Darier disease
Source: JCI Insight. 2023 Sep 22;8(18):e170739. doi: 10.1172/jci.insight.170739 (PMC10561730; doi:10.1172/jci.insight.170739)
Supplement: Supplemental data [file jciinsight-8-170739-s126.pdf]

Figure S1

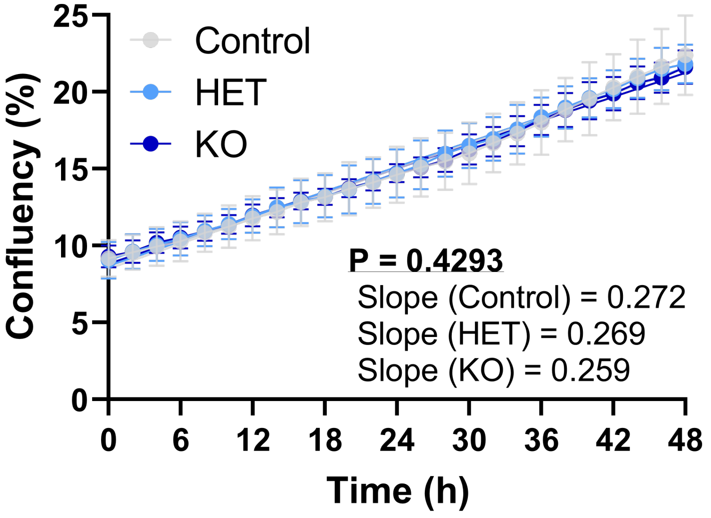

Depletion of SERCA2 does not alter proliferation of keratinocytes: *ATP2A2* control (+/+), HET (+/-), and KO (-/-) keratinocyte growth was measured over 48 hrs. Graph displays the mean +/- SD of the percent confluency across 25 non-overlapping microscopic fields at each time point for N=6 biological replicates per genotype; P-value calculated by simple linear regression to test if the slopes (growth rate) of the best-fit lines for each genotype were significantly different.

Figure S2

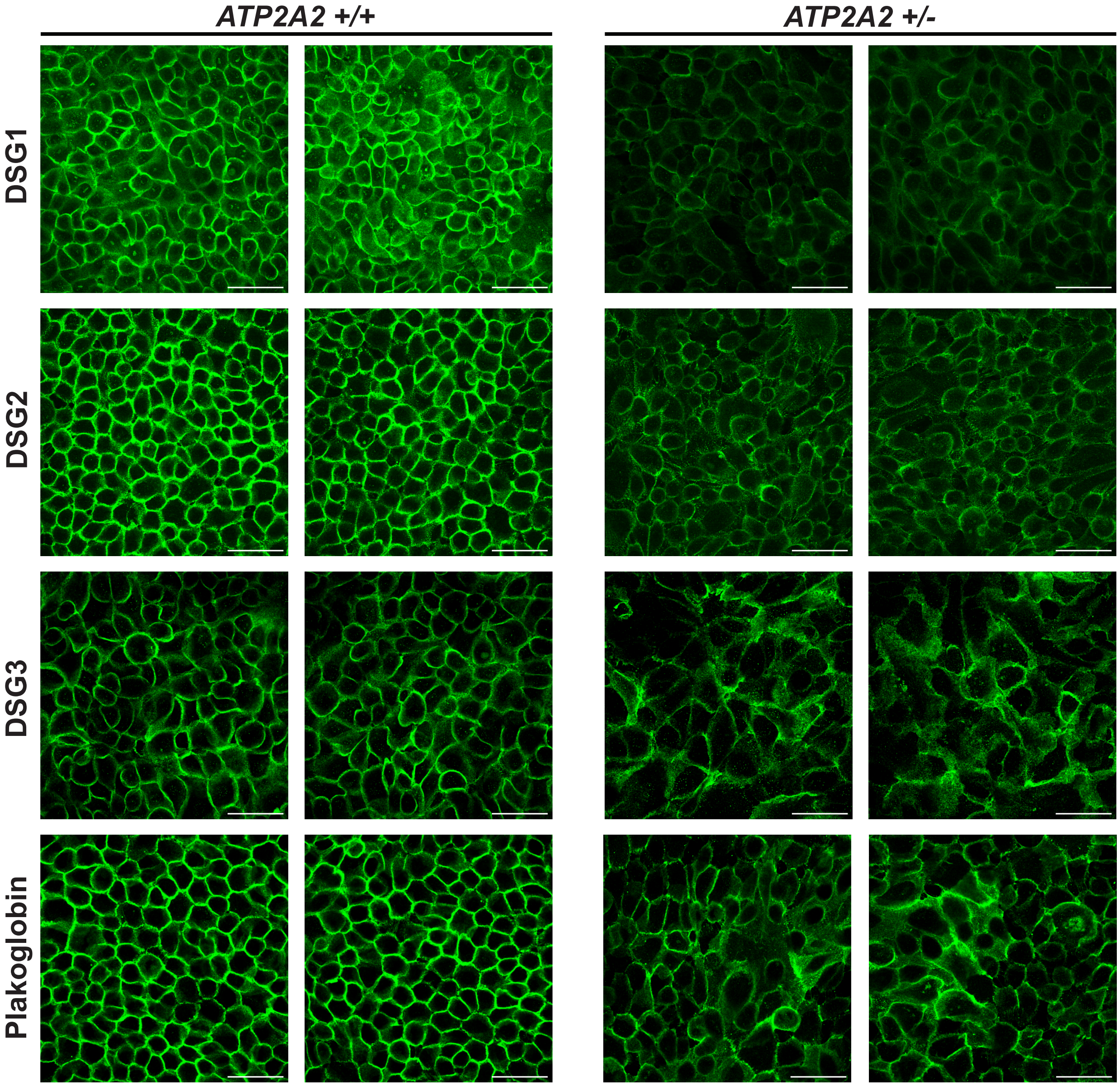

Desmosomal protein localization is impaired in SERCA2-deficient keratinocytes: *ATP2A2* control (+/+) and HET (+/-) cells were grown to confluency, then were switched into E-medium to induce differentiation and assembly of intercellular junctions. Immunostaining of desmosomal components after 24 hrs showed markedly reduced concentration of desmosomal cadherins (DSG1, DSG2, DSG3) and plakoglobin at sites of cell-cell contact in HET cells compared to control keratinocytes, which exhibited robust assembly of desmosomes. Images are representative of N=32 images from 2 experimental replicates; bar = 10 µm.

Figure S3

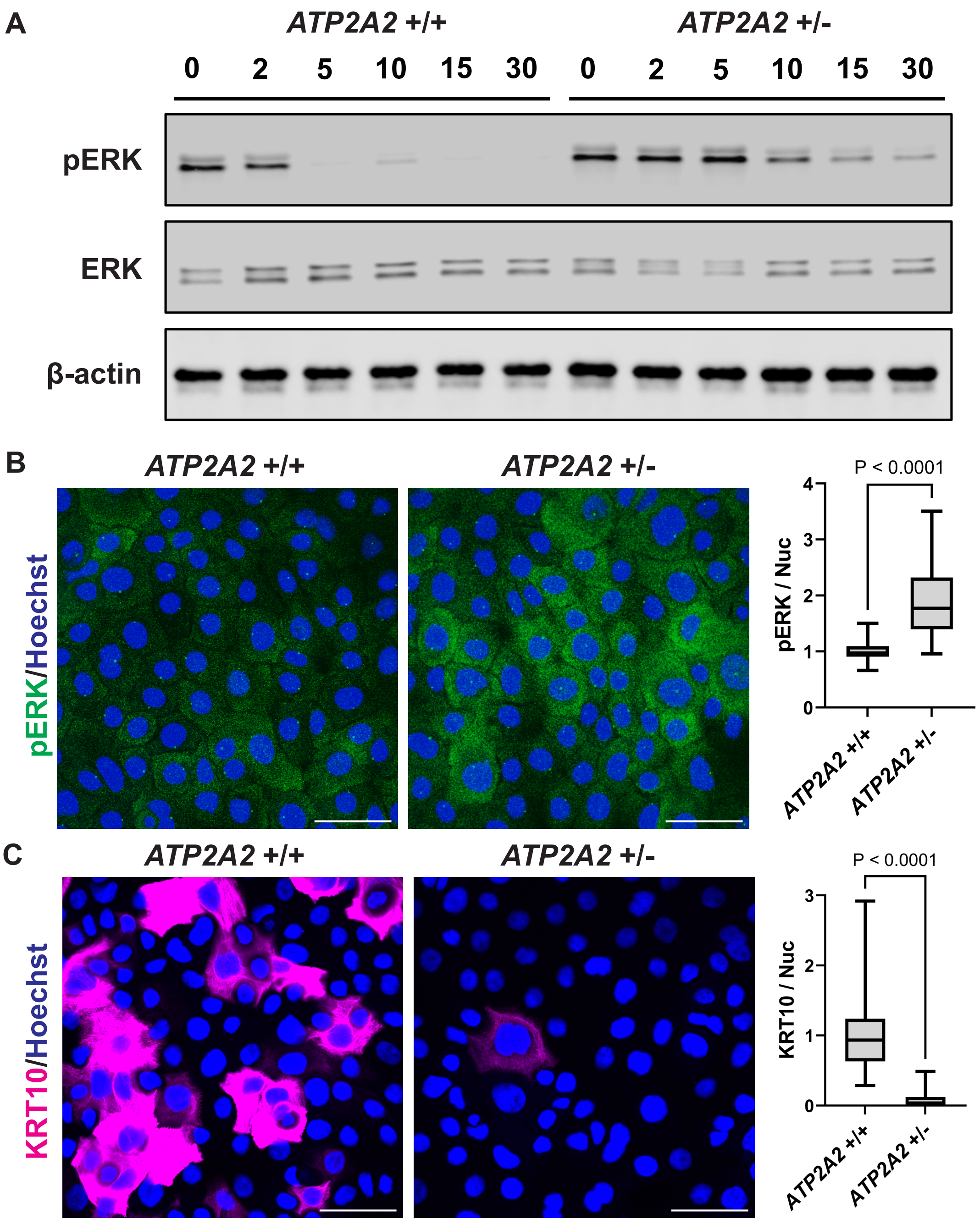

SERCA2-deficient keratinocytes exhibit aberrant ERK signaling and reduced KRT10 expression: **(A)** *ATP2A2* control (+/+) and HET (+/-) cells were grown to confluency, then were switched into medium lacking growth supplements and calcium for 4 hrs. Calcium was added to 1.3 mM and lysates were collected at baseline and after up to 30 min. Immunoblotting of pERK showed prolonged ERK activation in HET cells compared to controls; β-actin is a loading control. Blots are representative of 2 independent experiments. **(B, C)** *ATP2A2* control (+/+) and HET (+/-) cells were seeded at confluency, then were switched into E-medium to induce differentiation for 24 hrs. Fixed cells were immunostained for pERK or KRT10; Hoechst stains nuclei; bar = 10 μm. Fluorescence intensity data are shown as a box plot of the 25th-75th percentile with a line at the median from N≥47 non-overlapping high-powered microscopic fields per condition from 2 independent experiments; control mean normalized to 1; P-values are from two-tailed unpaired Student's t-test.
